# Erlanger Modell der Tinnitusentstehung – Perspektivwechsel und neue Behandlungsstrategie

**DOI:** 10.1007/s00106-023-01355-1

**Published:** 2023-09-15

**Authors:** Holger Schulze, Achim Schilling, Patrick Krauss, Konstantin Tziridis

**Affiliations:** Experimentelle HNO-Heilkunde, Hals-Nasen-Ohren-Klinik, Kopf- und Halschirurgie, Universitätsklinikum Erlangen, Waldstraße 1, 91054 Erlangen, Deutschland

**Keywords:** Stochastische Resonanz, Autokorrelation, Höroptimierung, Low intensity noise tinnitus suppression (LINTS), Rehabilitation, Stochastic resonance, Autocorrelation, Hearing optimization, Low intensity noise tinnitus suppression (LINTS), Rehabilitation

## Abstract

**Hintergrund:**

Etwa ein Sechstel der Bevölkerung westlicher Industrienationen leidet unter chronischem subjektivem Tinnitus, der allein in Deutschland volkswirtschaftliche Behandlungs- und Folgekosten von fast 22 Mrd. € pro Jahr verursacht. Nach der vorherrschenden Auffassung entsteht Tinnitus als Folge eines durch Hörverlust ausgelösten maladaptiven neurophysiologischen Prozesses im Gehirn.

**Ziel der Arbeit:**

Mit dem hier vorgelegten Erlanger Modell der Tinnitusentstehung wird ein umfassender neurophysiologischer Erklärungsansatz für das initiale Auftreten des Phantomgeräuschs nach Hörverlust vorgeschlagen. Auf der Grundlage des Modells wird eine neue Behandlungsstrategie entwickelt.

**Material und Methoden:**

Das hier zusammenfassend vorgestellte Modell beruht auf verschiedenen tier- und humanphysiologischen Untersuchungen der letzten Jahre.

**Ergebnisse:**

Das Erlanger Modell betrachtet subjektiven Tinnitus als Nebeneffekt eines physiologischen Mechanismus, der die Informationsübertragung in das auditorische System mittels stochastischer Resonanz (SR) auch im gesunden Hörsystem permanent optimiert. Tatsächlich hören hörgeschädigte Patienten mit Tinnitus im Mittel besser also solche ohne Tinnitus. Diese ungewohnte Perspektive auf das Phantomperzept kann betroffenen Patienten bereits dabei helfen, besser mit ihrem Leiden zurechtzukommen. Zusätzlich wurde, basierend auf dem Modell, als neue, individuell angepasste Behandlungsstrategie für tonalen Tinnitus die „low-intensity noise tinnitus suppression“ (LINTS) entwickelt und bereits erfolgreich an Patienten getestet.

**Schlussfolgerung:**

Möglicher limitierender Faktor für Modell und Behandlungsstrategie ist die Tonhöhe des Tinnitusperzepts, die es für Frequenzen über rund 5 kHz nötig machen könnte, Anpassungen an der Behandlungsstrategie vorzunehmen.

## Abstract (en)

**Background:**

About one sixth of the population of western industrialized nations suffers from chronic, subjective tinnitus, causing socioeconomic treatment and follow-up costs of almost 22 billion euros per year in Germany alone. According to the prevailing view, tinnitus develops as a consequence of a maladaptive neurophysiological process in the brain triggered by hearing loss.

**Objectives:**

The Erlangen model of tinnitus development presented here is intended to propose a comprehensive neurophysiological explanation for the initial occurrence of the phantom sound after hearing loss. Based on the model, a new treatment strategy will be developed.

**Materials and methods:**

The model summarized here is based on various animal and human physiological studies conducted in recent years.

**Results:**

The Erlangen model considers subjective tinnitus as a side effect of a physiological mechanism that permanently optimizes information transmission into the auditory system by means of stochastic resonance (SR) even in the healthy auditory system. In fact, hearing-impaired patients with tinnitus hear better on average than those without tinnitus. This unfamiliar perspective on the phantom percept may already help affected patients to cope better with their suffering. In addition, based on the model, low intensity noise tinnitus suppression (LINTS) has been developed as a new, individually adapted treatment strategy for tonal tinnitus and has already been successfully tested in patients.

**Conclusions:**

A possible limiting factor for the model and treatment strategy is the pitch of the tinnitus percept, which may require adjustments to the treatment strategy for frequencies above about 5 kHz.

Das auf stochastischer Resonanz (SR) basierende Erlanger Modell der Tinnitusentstehung betrachtet das Phantomperzept nicht als rein pathophysiologisches Phänomen, sondern als Nebenprodukt eines physiologischen Mechanismus, der dazu dient, das Hören kontinuierlich zu optimieren. Neben dieser ungewohnten Sichtweise auf das Leiden lässt sich aus dem Modell auch eine neue, auf den einzelnen Patienten zugeschnittene Behandlungsstrategie ableiten, welche das Ziel hat, das Tinnitusperzept zu unterdrücken und sich zumindest bei einer Subgruppe von Patienten bereits als erfolgversprechend gezeigt hat.

In westlichen Industrienationen leidet etwa ein Sechstel der Bevölkerung unter chronischem subjektivem Tinnitus [20], und allein in Deutschland verursacht Tinnitus volkswirtschaftliche Behandlungs- und Folgekosten von fast 22 Mrd. € pro Jahr [25]. Im Gegensatz zur vorherrschenden Auffassung, welche Tinnitus als Folge eines durch einen Hörverlust ausgelösten maladaptiven Prozesses betrachtet, stellten die Autoren vor einigen Jahren ein alternatives Modell der Tinnitusentwicklung vor, welches die Phantomwahrnehmung als Nebeneffekt eines physiologischen Mechanismus betrachtet, der die Informationsübertragung in das auditorische System mittels stochastischer Resonanz (SR) auch im gesunden Hörsystem permanent optimiert [8–10, 19]. Dieser mechanistische Erklärungsansatz für das Auftreten eines Tinnitusperzepts ermöglichte die Entwicklung einer neuen Behandlungsstrategie, der „low-intensity noise tinnitus suppression“ (LINTS), welche sich bereits in 2 Pilotstudien als erfolgversprechend herausgestellt hat [17, 23]. Im Folgenden erläutern die Autoren, ausgehend von diesem Erlanger Modell, diese neue Behandlungsstrategie und diskutieren abschließend mögliche Limitationen und Weiterentwicklungsmöglichkeiten.

## Das Erlanger Modell der Tinnitusentstehung

Wie die meisten Modelle geht auch das der Autoren davon aus, dass initial ein Hörverlust ursächlich für die Entstehung eines Tinnitusperzepts ist. Der Hörverlust beruht dabei auf einer Reduktion der Innervation der inneren Haarzellen der Cochlea [24], welche so gering sein kann, dass im Audiogram noch keine Erhöhung der Hörschwellen gemessen werden kann („hidden hearing loss“, [12]) oder nur eine klinisch nicht relevante, die noch keiner Versorgung mit einer Hörhilfe bedarf.

Nach dem Erlanger Modell kann es keine Tinnitusentstehung ohne zumindest geringfügigen Hörverlust geben

Im Ergebnis resultiert allerdings dennoch ein reduzierter Input aus der Cochlea in den dorsalen Nucleus cochlearis (DCN), welcher dort bereits dazu führen kann, dass schwache Signale keine überschwellige Reaktion der entsprechenden Neurone im DCN mehr auslösen und somit auch nicht mehr in die nachfolgende Hörbahn weitergeleitet würden [2]. Dies kann sich z. B. in einer verschlechterten Sprachwahrnehmung bei Umgebungsgeräuschen ausdrücken. Nach dem Erlanger Modell kann es also keine Tinnitusentstehung ohne einen zumindest geringfügigen Hörverlust in der beschriebenen Art geben. Dies könnte auch ein transienter Hörverlust sein, etwa nach einem Konzertbesuch, der dann auch nur zu einem transienten Tinnitus führen würde. Die Frage, ob es jenseits des Erklärungsspektrums dieses Modells eine Tinnitusentstehung geben kann, etwa durch Stress, untersuchen die Autoren derzeit in ihrem Labor, halten dies aber für unwahrscheinlich.

**Figure 1:**
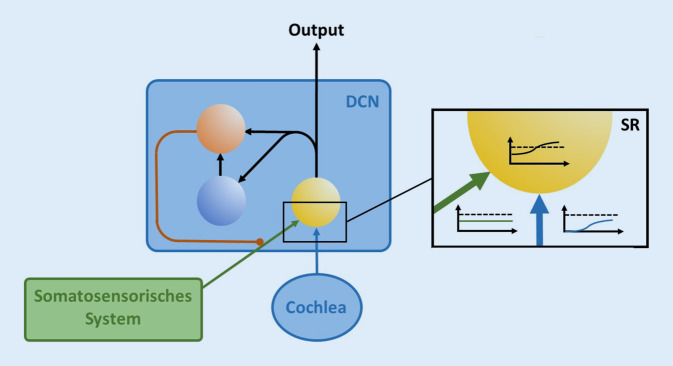

Das vorliegende Modell (Abb. 1) postuliert nun, dass auf der Ebene der DCN-Neurone (Abb. 1, gelbes Neuron) das Phänomen der sog. SR (Abb. 1 Ausschnittsvergrößerung) stattfindet, um ebendiese Übertragung schwacher Signale in die Hörbahn dennoch zu ermöglichen und so das Hören insgesamt zu verbessern. Dabei wird dem schwachen Eingangssignal aus der Cochlea (Abb. 1, blaue Pfeile) ein Rauschen in Form von neuronaler Spontanaktivität (Abb. 1, grüne Pfeile) hinzuaddiert, sodass die Summe aus Eingangssignal und Rauschen groß genug ist, um das DCN-Neuron überschwellig zu aktivieren und somit die Weiterleitung des Signals zu ermöglichen (Abb. 1 Ausschnittsvergrößerung). Die Quelle für das neuronale Rauschen ist dabei hochwahrscheinlich im somatosensorischen System verortet [3, 11, 22], was z. B. auch erklärt, warum viele Patienten ihr Ohrgeräusch durch Anspannen der Kiefermuskulatur modulieren können. Auch Phänomene wie somatischer Tinnitus [21] oder die Verbesserung des Sprachverständnisses von Cochleaimplantat(CI)-Patienten bei elektrotaktiler Stimulation der Fingerspitzen [6] können dadurch erklärt werden.

Die Diagramme in Abb. 1 zeigen verschiedene Ratenintensitätsfunktionen: Mit steigender Lautstärke (blaue Kurve) eines externen Schallsignals (Abszisse) steigt die Aktivität des cochleären Inputs (Ordinate), erreicht aber nicht die Schwelle des DCN-Neurons (gestrichelte Linie). Der Input aus dem somatosensorischen System ist unabhängig von der Lautstärke des Schallsignals (grüne Kurve, der somatosensorische Input ist vereinfachend als konstanter Wert dargestellt. Allerdings handelt es sich in Realität um unkorreliertes Rauschen. Der hier gezeigt konstante Wert kann als Rauschamplitude betrachtet werden). Die Summe aus somatosensorischem und cochleärem Input weist den gleichen dynamischen Bereich auf wie der cochleäre Input und wird ab einer bestimmten Lautstärke des Schallsignals überschwellig (schwarze Kurve).

Damit SR wie in Abb. 1 beschrieben der Optimierung der Informationsübertragung in das auditorische System dienen kann, muss die Amplitude des Rauschens (Abb. 1, grün) so gewählt sein, dass der Informationsgehalt des DCN-Outputs maximal wird. Ist diese Amplitude zu gering, erreicht auch die Summe von cochleärem und somatosensorischem Input nicht die Schwelle der DCN-Neurone, ist sie zu groß, geht das Signal im Rauschen unter. Nach dem Modell der Autoren berechnet der DCN zur Bestimmung dieser optimalen Rauschamplitude die Autokorrelation des DCN-Outputs, welche ein direktes Maß für dessen Informationsgehalt ist [8], und optimiert diesen Informationsgehalt mittels eines Regelkreises. Hierzu nutzt der DCN „delay lines“ [13], welche ein Signal zeitverzögert mit sich selbst überlagern und so regelmäßig auftretende Muster in festen zeitlichen Abständen erkennen können (welche im Rauschen so nicht zu erwarten sind). Wie in Abb. 1 erkennbar, wird dazu der Output des gelben Neurons einerseits direkt und andererseits mit einer dem zu detektierenden Zeitintervall entsprechenden frequenzspezifischen Zeitverzögerung (über das blaue Delay-Neuron) auf einen Koinzidenzdetektor (rotes Neuron) geleitet. Dieser reagiert nur dann, wenn er gleichzeitig Input aus der direkten und der zeitverzögerten Projektion erhält, also genau dann, wenn 2 Aktionspotenziale mit dem gewünschten zeitlichen Abstand auftreten, welcher der Eigenfrequenz im jeweiligen Frequenzkanal entspricht. Bei einer Eigenfrequenz von beispielsweise 1 kHz etwa betrüge dieses Intervall also 1 ms.

Der Koinzidenzdetektor schließlich hemmt nun seinerseits den somatosensorischen Input [3]. Je größer der Informationsgehalt des DCN-Outputs ist, desto weniger Rauschen muss aus dem somatosensorischen System dem cochleären Input hinzuaddiert werden, um eine optimale Informationsübertragung zu gewährleisten. So schließt sich der Regelkreis.

Die Wahrnehmung eines Tinnitus entsteht, wenn das beigemischte Rauschen selbst überschwellig ist

**Figure 2:**
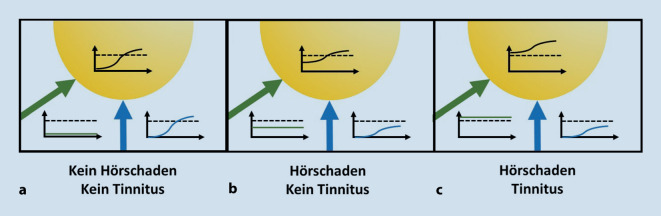

Die Wahrnehmung eines Tinnitus entsteht in diesem Modell immer dann, wenn das beigemischte Rauschen aus dem somatosensorischen System selbst bereits überschwellig ist (Abb. 2). Das heißt, das dem cochleären Input für SR aus dem somatosensorischen System beigemischte Rauschen dient nicht nur der Optimierung der Informationsübertragung ins Hörsystem, es ist auch die Quelle der dem Tinnitus zugrunde liegenden neuronalen Hyperaktivität.

In Abb. 2a ist die Situation dargestellt, in der kein Hörschaden vorliegt. Die Übertragung der Signale aus der Cochlea (blau) ist auch ohne Beimischung von neuronalem Rauschen aus dem somatosensorischen System (grün) optimal. Es entsteht kein Tinnitus. In Abb. 2b wird die Situation gezeigt, in der ein Hörschaden vorliegt, aber die Amplitude des beigemischten Rauschens allein wird im DCN nicht überschwellig. Die Hörschwelle wird etwas verbessert, es entsteht aber kein Tinnitus. Dagegen liegt in der Situation in Abb. 2c ein Hörschaden vor, und die Amplitude des beigemischten Rausches allein ist bereits überschwellig, d. h., es kommt zur Aktivierung der Neurone ohne externen Reiz, was als Tinnitus wahrgenommen wird. Die Hörschwelle wird dabei nochmal besser.

Selbstverständlich existieren neben diesem Modell zur Tinnitusentstehung noch weitere, wie etwa das neurophysiologische Tinnitusmodell nach Jastreboff oder die Modelle der thalamokortikalen Dysrhythmie, homöostatischen Plastizität, lateraler Inhibition und anderer, deren Diskussion allerdings den Rahmen dieses Artikels sprengen würde. Diese haben die Autoren einander aber an anderer Stelle ausführlichst gegenübergestellt [8, 17, 18].

Nach dem Erlanger Modell betrachten die Autoren Tinnitus also als Begleiterscheinung eines Mechanismus, der eigentlich dazu da ist, das Hören permanent und schnell (im Millisekundenbereich) an sich verändernde Hörsituationen anzupassen. Dabei ist zu beachten, dass die Beimischung von Rauschen optimaler Amplitude das Hören grundsätzlich verbessert (bis zu einer unphysiologischen Grenze, bei der das Rauschen das Signal überdecken würde). Tinnitus ist jedoch gewissermaßen der Preis, den das Hörsystem dafür zahlt. Tatsächlich konnten die Autoren diese Verbesserung des Hörens bei Tinnituspatienten im Vergleich zu Patienten ihrer HNO-Klinik ohne Tinnitus bereits nachweisen [4]. Hierbei ist jedoch einschränkend darauf hinzuweisen, dass sich die Hörverbesserung der Patienten ohne Tinnitus nicht von der eines hypothetischen Patienten ohne SR unterscheiden lässt; man kann nur den Nutzen für die Hörschwelle der Tinnituspatienten relativ zu den Patienten ohne Tinnitus bestimmen. Dieser Perspektivwechsel auf das Phänomen Tinnitus, die Erkenntnis, dass Tinnitus neben dem Leiden, das er auslöst, auf Mechanismen beruht, die letztlich der Verbesserung des Hörens dienen, kann Patienten nach Erfahrung der Autoren bereits helfen, besser mit ihrem Tinnitus umzugehen. Doch selbstverständlich ist das noch nicht genug.

## „Low-intensity noise tinnitus suppression“

Auf der Grundlage des beschriebenen Erlanger Modells der Tinnitusentstehung haben die Autoren nun eine neue Strategie zur Behandlung von Tinnituspatienten entwickelt: „low-intensity noise tinnitus suppression“ (LINTS).

**Figure 3:**
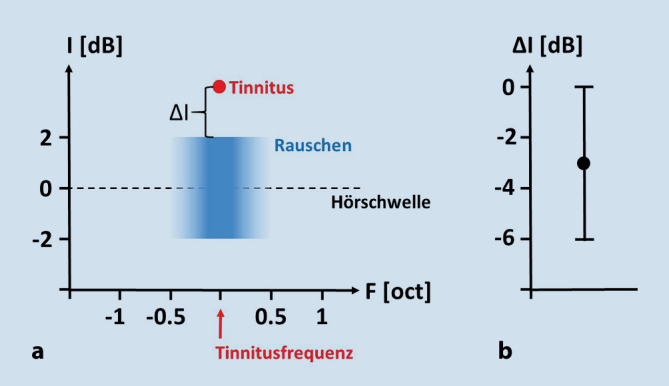

Die Behandlung basiert auf der Grundidee, das interne neuronale Rauschen, welches wie dargestellt der Verbesserung des Hörens mittels SR dient, aber als Tinnitus wahrgenommen werden kann, durch schwellennahes externes akustisches Rauschen zu ersetzen. In diesem Fall würde das externe Rauschen das Hören über SR verbessern und es überflüssig machen, internes Rauschen aus dem somatosensorischen System beizumischen. Mehr noch, ist die optimale Amplitude und spektrale Zusammensetzung des externen Rauschens individuell für einen bestimmten Patienten erst einmal gefunden, würde der beschriebene Regelkreis das Rauschen aus dem somatosensorischen System sogar aktiv unterdrücken, da die Autokorrelation des DCN-Outputs dann bereits maximiert und damit die Hemmung des somatosensorischen Inputs durch das Koinzidenzneuron maximal wäre. Ermutigt, dass dieser Ansatz prinzipiell funktionieren sollte, wurden die Autoren dabei durch eine Studie von Zeng et al. [26], die zeigten, dass die Präsentation von schwellennahem externem Rauschen bei normalhörenden Probanden die Hörschwellen zu verbessern vermag. Mittlerweile konnten die Autoren in 2 Pilotstudien den prinzipiellen Nachweis erbringen, dass sich durch ein geeignetes externes Rauschen tatsächlich die Lautstärke des von Patienten wahrgenommenen Tinnitus reduzieren und unter optimalen Bedingungen sogar ganz unterdrücken lässt [17, 23]. Dabei sei an dieser Stelle darauf hingewiesen, dass das eingesetzte Rauschen schwellennah und daher keinesfalls in der Lage ist, den wahrgenommenen Tinnitus zu maskieren (Abb. 3b). Es wird also nicht das eine belastende Geräusch durch ein anderes ersetzt. Zum anderen müssen die Patienten natürlich über ein noch ausreichendes Hörvermögen im geschädigten Ohr verfügen, damit das extern eingespielte Rauschen überhaupt aufgenommen und an den DCN weitergeleitet werden kann. Hier hat sich eine Grenze von maximal 40 dB Hörverlust als limitierender Faktor herausgestellt. Darüber hinaus haben die Autoren bislang nur Patienten mit tonalem Tinnitus, bei welchen sich die Tinnitusfrequenz über ein einfaches Ton-Matching-Verfahren gut bestimmen ließ, in die Studien eingeschlossen.

„Low-intensity noise tinnitus suppression“ ist eine neue Strategie zur Behandlung von Tinnitus

Als besonders wirkungsvoll erwies sich in dieser Patientengruppe folgender Parameterbereich für das externe Rauschen (Abb. 3): Die Patienten erlebten die stärkste Reduktion der Tinnituslautstärke – und in Einzelfällen eine Auslöschung des Tinnitus – bei Stimulation mit Schmalbandrauschen mit einer Mittenfrequenz, die der individuellen Tinnitusfrequenz des Patienten entsprach, und einem gaußförmigen Spektrum von einer Oktave Breite. Die Lautstärke dieses optimalen Rauschens bewegte sich in der Regel im Bereich von ±2 dB um die Hörschwelle des Patienten (blauer Bereich in Abb. 3a). Der Median der Lautstärke des Rauschens relativ zur empfundenen Tinnituslautstärke lag bei −3 dB, war also meist leiser als das Tinnitusperzept selbst (Abb. 3b). Eine Maskierung des Tinnitus durch das Rauschen fand also nicht statt.

## Grenzen und Horizonte

Wie beschrieben wird in diesem Modell zur Abschätzung des in das auditorische System weitergeleiteten Informationsgehalts die Autokorrelation des DCN-Outputs berechnet. Die Information wird somit immer dann in der zeitlichen Abfolge von Aktionspotenzialen erkannt, wenn die Zeitabstände zwischen den einzelnen Aktionspotenzialen nicht völlig zufällig verteilt sind, sondern die Intervalle der entsprechenden Eigenfrequenz des jeweiligen Frequenzkanals enthalten. Offensichtlich erfordert die Berechnung der Autokorrelation ein präzises Timing der Aktionspotenziale, d. h. eine Phasenkopplung der Aktionspotenziale an die Wellenform oder Umhüllende des Reizes [16]. Bei Säugetieren ist eine solche Phasenkopplung (im Hörnerv) selbst mit Hilfe des Wever-Salvenprinzips aber nur für Frequenzen bis zu etwa 5 kHz möglich [5, 14]. Wenn also die Optimierung des beschriebenen SR-Mechanismus auf der Berechnung der Autokorrelation des DCN-Ausgangs beruht und die Berechnung dieser Autokorrelation von einem präzisen Timing der Aktionspotenziale abhängt, dann sollte die beobachtete Verbesserung der Hörschwelle nur für Frequenzen bis zu etwa 5 kHz möglich sein.

Die wahrgenommene Tinnitusfrequenz korreliert stark mit der Frequenz des stärksten Hörverlusts

**Figure 4:**
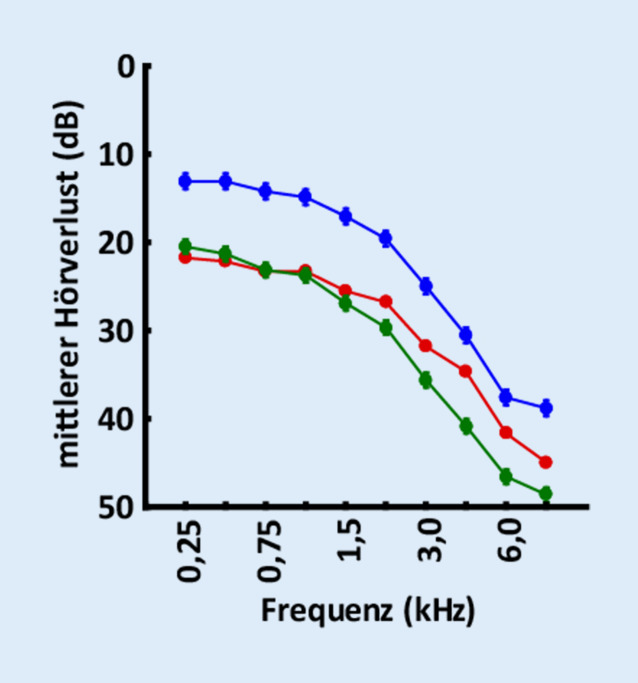

In Übereinstimmung mit diesem Modell korreliert die von Patienten wahrgenommene Tinnitusfrequenz stark mit der Frequenz des stärksten Hörverlusts [1, 7, 15], da dies der Frequenzbereich ist, in welchem dem DCN die höchsten Geräuschpegel zugeführt werden müssten, um eine optimale SR und damit eine maximale Schwellenverbesserung zu gewährleisten. Demnach müssten Patienten mit Tinnitusfrequenzen bis zu 5 kHz im Vergleich zu Patienten ohne Tinnitus eine verbesserte Hörschwelle aufweisen, während Patienten mit Tinnitusfrequenzen über 5 kHz keine solche Verbesserung zeigen sollten. Dies ist tatsächlich der Fall (Abb. 4).

In Abb. 4 ist die Abhängigkeit der Hörschwellenverbesserung von der Tinnitusfrequenz dargestellt. Patienten mit Tinnitusfrequenzen bis zu 5 kHz (blau) weisen im Vergleich zu Patienten ohne Tinnitus (rot) verbesserte Hörschwellen auf, während Patienten mit Tinnitusfrequenzen über 5 kHz (grün) keine solche Verbesserung zeigen. Die Daten wurden aus der audiologischen Datenbank der HNO-Klinik, Kopf- und Halschirurgie Erlangen gewonnen. Hierzu wurden anonymisierte klinische Standard-Reintonaudiometrie-Daten (Hörverlust, HL, in dB; Tinnitustonhöhe, TP) von beiden Ohren von 47.986 erwachsenen Patienten (0,25 bis 8 kHz), die zwischen 2000 und 2018 gesammelt wurden, retrospektiv analysiert. Daher war nach deutschem Recht keine Einverständniserklärung erforderlich. Patienten, die über tonale Tinnituswahrnehmungen in mindestens einem Ohr klagten, wurden als Tinnituspatienten (Gruppe T) und Patienten ohne Beschwerden über irgendeine Form von Tinnitus als Daten von Nichttinnituspatienten (Gruppe NT) eingestuft. Patienten, die über nichttonalen Tinnitus klagten, wurden von der weiteren Analyse ausgeschlossen. Die Gruppe T wurde weiter nach der wahrgenommenen TP unter oder über 5 kHz eingeteilt (eine TP von 5 kHz konnte aufgrund der im klinischen TP-Testraster verwendeten Frequenzen, die 5 kHz ausschlossen, nicht auftreten). Die Daten von insgesamt 44.050 NT-, 1833 „TP < 5 kHz“- und 2103 „TP > 5 kHz“-Patienten wurden durch eine 2‑faktorielle ANOVA und Tukey-Post-hoc-Tests analysiert. Diese Tests ergaben, dass die „TP < 5 kHz“-Gruppe im Vergleich zu den „TP > 5 kHz“- und NT-Gruppen bei allen getesteten Frequenzen deutlich weniger von Hörverlust betroffen war. Die „TP > 5 kHz“- und NT-Gruppen hingegen zeigten einen ähnlichen mittleren Hörverlust für niedrigere Frequenzen bis zu 1,5 kHz. Ab 2 kHz zeigten die „TP > 5 kHz“-Gruppen signifikant schlechtere mittlere Hörschwellen im Vergleich zur NT-Gruppe.

Dieses in seiner Klarheit überraschende Ergebnis stützt die Hypothese der Autoren, dass die SR-basierte Optimierung der Informationsübertragung in das auditorische System auf der Berechnung der Autokorrelationsfunktion des DCN-Outputs beruht. Man beachte dabei, dass die Schwellenverbesserung nach diesem Modell zwar auf den betroffenen Frequenzbereich eines einzelnen Patienten beschränkt sein sollte, d. h. maximal im Frequenzbereich der Tinnitusfrequenz, dass aber die mittlere Verbesserung über alle Patienten zu einer mehr oder weniger parallelen Verschiebung des mittleren Audiogramms der Gruppe „TP < 5 kHz“ im Vergleich zu den mittleren Audiogrammen der NT- bzw. „TP > 5 kHz“-Gruppe führen sollte (Abb. 4). Die Tinnitusgruppe mit einer Tinnitusfrequenz oberhalb von 5 kHz, die nicht von dem vorgeschlagenen SR-Mechanismus profitieren konnte, zeigte im Frequenzbereich oberhalb von etwa 2 kHz im Durchschnitt sogar schlechtere Hörschwellen als die Gruppe NT, was auf einen Maskierungseffekt des von den Patienten wahrgenommenen Tinnitus in diesem Frequenzbereich zurückzuführen sein könnte.

Bleibt die Frage, ob auch die von den Autoren vorgeschlagene Behandlungsstrategie LINTS von diesem Befund betroffen ist; ob die Methode also bei Patienten mit Tinnitusfrequenzen oberhalb von rund 5 kHz weniger gut oder sogar gar nicht mehr funktioniert. Dies wird aktuell im Labor der Autoren untersucht. Sollte sich tatsächlich herausstellen, dass Patienten mit Tinnitusfrequenzen oberhalb von rund 5 kHz nicht mehr von LINTS profitieren können, da die fehlende zeitliche Kodierung des hochfrequenten Signals keine positive Autokorrelation des DCN-Outputs mehr ermöglicht, wollen die Autoren eine Modifikation von LINTS testen, bei der das eingesetzte Rauschen seinerseits zeitlich amplitudenmoduliert wird, um so wieder eine positive Autokorrelation auch in den hohen Frequenzkanälen zu ermöglichen.

## Fazit für die Praxis

- Nach dem Erlanger Modell der Tinnitusentstehung ist subjektiver Tinnitus kein rein pathophysiologisches Phänomen, sondern Nebenprodukt eines physiologischen Mechanismus, der dazu dient, das Hören kontinuierlich zu optimieren.
- Tatsächlich hören hörgeschädigte Patienten mit Tinnitus im Mittel besser also solche ohne Tinnitus.
- Diese ungewohnte Perspektive auf das Phantomperzept kann betroffenen Patienten bereits dabei helfen, besser mit ihrem Leiden zurechtzukommen.
- Auf der Grundlage dieses Modells wurde mit der „low-intensity noise tinnitus suppression“ (LINTS) eine neue Strategie zur Behandlung von Tinnituspatienten entwickelt.
- Diese basiert auf der Grundidee, dass internes neuronales Rauschen, welches im Modell der Verbesserung des Hörens dient, aber als Tinnitus wahrgenommen werden kann, durch schwellennahes, externes, individuell angepasstes akustisches Rauschen ersetzt wird.
- Eine mögliche Limitierung des Modells und der Behandlungsmethode stellen Patienten mit einer Tinnitusfrequenz von über 5 kHz dar. Dies wird aktuell untersucht.
